# Ecology and potential functions of plant-associated microbial communities in cold environments

**DOI:** 10.1093/femsec/fiab161

**Published:** 2021-12-15

**Authors:** Malek Marian, Giorgio Licciardello, Bianca Vicelli, Ilaria Pertot, Michele Perazzolli

**Affiliations:** Center Agriculture Food Environment (C3A), University of Trento, via E. Mach 1, 38098 San Michele all'Adige, Italy; Research and Innovation Centre, Fondazione Edmund Mach, via E. Mach 1, 38098 San Michele all'Adige, Italy; Center Agriculture Food Environment (C3A), University of Trento, via E. Mach 1, 38098 San Michele all'Adige, Italy; Research and Innovation Centre, Fondazione Edmund Mach, via E. Mach 1, 38098 San Michele all'Adige, Italy; Center Agriculture Food Environment (C3A), University of Trento, via E. Mach 1, 38098 San Michele all'Adige, Italy; Research and Innovation Centre, Fondazione Edmund Mach, via E. Mach 1, 38098 San Michele all'Adige, Italy; Center Agriculture Food Environment (C3A), University of Trento, via E. Mach 1, 38098 San Michele all'Adige, Italy; Research and Innovation Centre, Fondazione Edmund Mach, via E. Mach 1, 38098 San Michele all'Adige, Italy; Center Agriculture Food Environment (C3A), University of Trento, via E. Mach 1, 38098 San Michele all'Adige, Italy; Research and Innovation Centre, Fondazione Edmund Mach, via E. Mach 1, 38098 San Michele all'Adige, Italy

**Keywords:** plant microbiota, cold environments, cold stress, beneficial microbial communities, cold tolerance

## Abstract

Complex microbial communities are associated with plants and can improve their resilience under harsh environmental conditions. In particular, plants and their associated communities have developed complex adaptation strategies against cold stress. Although changes in plant-associated microbial community structure have been analysed in different cold regions, scarce information is available on possible common taxonomic and functional features of microbial communities across cold environments. In this review, we discuss recent advances in taxonomic and functional characterization of plant-associated microbial communities in three main cold regions, such as alpine, Arctic and Antarctica environments. Culture-independent and culture-dependent approaches are analysed, in order to highlight the main factors affecting the taxonomic structure of plant-associated communities in cold environments. Moreover, biotechnological applications of plant-associated microorganisms from cold environments are proposed for agriculture, industry and medicine, according to biological functions and cold adaptation strategies of bacteria and fungi. Although further functional studies may improve our knowledge, the existing literature suggest that plants growing in cold environments harbor complex, host-specific and cold-adapted microbial communities, which may play key functional roles in plant growth and survival under cold conditions.

## Teaser

Review on plant-associated microbial communities in alpine, Arctic and Antarctic regions: factors affecting the taxonomic structure: discussion on dominant taxa and their possible functional properties.

## INTRODUCTION

Cold environments are characterized by average daily air temperatures below 5°C throughout the year and are located in specific areas of the Earth's biosphere, such as alpine and polar (Arctic and Antarctica) regions (Zakhia *et al*. 2008). The term ‘alpine’ is used in this review to indicate regions with high elevation mountains, that include not only the Alps, but the mountain areas of Europe, Asia (e.g. Hindu Kush, Karakorum–Himalaya and Tibetan Plateau) and America (e.g. Rocky Mountains and South American Alps; Casanueva *et al*. 2010). Arctic regions are defined by the Arctic Circle, which include continental lands in northern Asia (e.g. Siberia), Europe (e.g. Scandinavia), North America (e.g. Alaska and northern Canada) and islands, such as Novaya Zemlya (Russia), Svalbard (Norway), Iceland and Southern Greenland (Denmark). Antarctic regions include the Antarctic continent and sub-Antarctic islands (Convey *et al*. 2014). Although alpine and Arctic environments have some similarities (e.g. short growing seasons with low temperatures available for plants, soils with low levels of nutrients), they are characterized by distinct features (Ives and Barry 2019). In particular, extreme wind speeds, high snowfall and well-drained soils are typically found in the alpine environments, while high annual fluctuations of solar radiation, moderate winds, low snowfall and water-logged soils due to underlying permafrost characterize Arctic environments (Ives and Barry 2019). On the other hand, the Antarctic is the coldest and driest region of the world, and it is considered among the most limiting and stressful environments for plant life (Convey *et al*. 2014).

Vegetation in cold regions comprises less than 7% (ca. 10 million km^2^) of the Earth's terrestrial surface (Breen *et al*. 2014; Lee *et al*. 2017). In alpine and Arctic areas, vascular plants (including angiosperms) are prevalent below the latitudinal and altitudinal tree lines, which correspond to the limit of forest where trees naturally do not persist (Breen *et al*. 2014). On the other hand, only two angiosperms, namely *Colobanthus quitensis* and *Deschampsia antarctica*, can grow in the Antarctic environments (Convey 2013).

Plants are associated with complex microbial communities, whose size and taxonomic structure depend on biotic (e.g. plant species, age and type of tissue) and abiotic factors (e.g. climatic conditions and soil physiochemical characteristics; Compant *et al*. 2019). Moreover, members of plant-associated microbial communities interact with the host plant providing neutral, detrimental or beneficial effects (Montesinos 2003). Increasing evidences support that microbial communities can promote plant growth at low temperatures and improve plant tolerance to cold stress (Acuña-Rodríguez *et al*. 2020). In this review, we summarize the current knowledge on plant-associated microbial communities in alpine, Arctic and Antarctic regions, in order to discuss key factors affecting the taxonomic structure of microbial communities in cold environments and to highlight the most abundant plant-associated taxa in terms of relative abundance (dominant taxa) and their possible functional properties in such environments.

## PLANT-ASSOCIATED MICROBIAL COMMUNITIES IN ALPINE REGIONS

**Figure 1.**
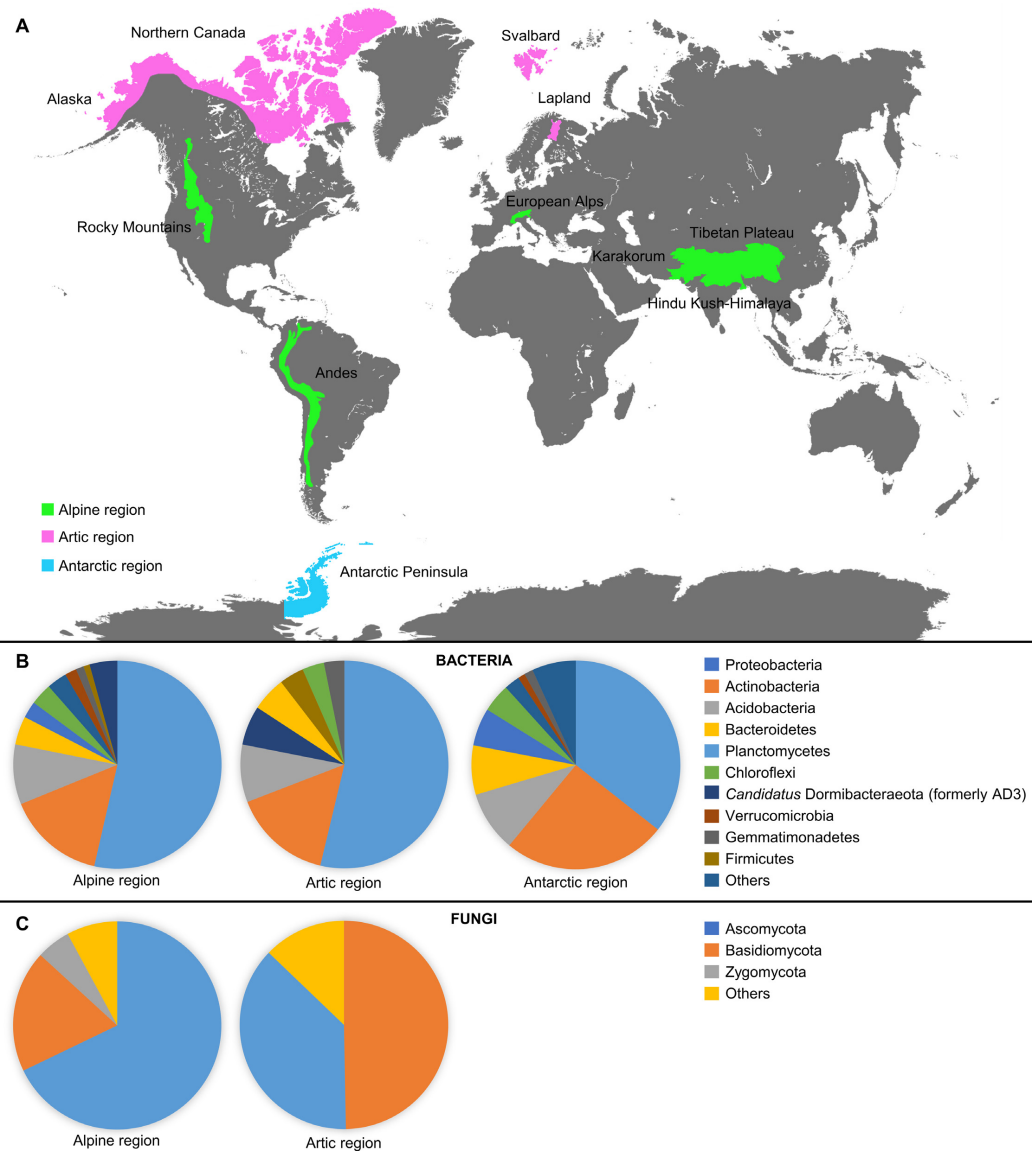
Geographical location **(A)** and taxonomy of plant-associated bacterial **(B)** and fungal **(C)** communities in alpine, Arctic and Antarctic regions. Plant-associated microbial communities were studied in European Alps (Garnica *et al*. 2013; Casazza *et al*. 2017; Kumar *et al*. 2017; Roy *et al*. 2018; Oberhofer *et al*. 2019; Praeg, Pauli and Illmer 2019; Wassermann *et al*. 2019), Hindu Kush, Karakorum and Himalaya–Tibetan Plateau (Bisht, Mishra and Joshi 2013; Li *et al*. 2014; Cui *et al*. 2015; Angel *et al*. 2016; Kotilínek *et al*. 2017; Chang *et al*. 2018; Jamil *et al*. 2020, 2018; Sheng *et al*. 2011; Pan *et al*. 2013; Řeháková *et al*. 2015; Lu *et al*. 2016; Wang *et al*. 2016; Ma *et al*. 2020), Andes (Calvo *et al*. 2010; Correa-Galeote *et al*. 2016; Jorquera *et al*. 2016; Ruiz-Pérez, Restrepo and Zambrano 2016; Pfeiffer *et al*. 2017; Castellano-Hinojosa *et al*. 2018; Chica *et al*. 2019; Chumpitaz-Segovia *et al*. 2020; Senés-Guerrero and Schüssler, 2016), Rocky Mountains, USA (Schmidt *et al*. 2008; Bueno de Mesquita *et al*. 2018), Lapland, Finland (Nissinen, Männistö and van Elsas 2012; Kauppinen *et al*. 2014; Kumar *et al*. 2017; Given *et al*. 2020), Svalbard, Norway (Botnen *et al*. 2014, 2020; Bjorbækmo *et al*. 2010; Öpik *et al*. 2013; Blaalid *et al*. 2014; Zhang and Yao 2015; Mundra, Bahram and Eidesen 2016; Kumar *et al*. 2017; Lorberau *et al*. 2017; Newsham *et al*. 2017), Northern Canada (Allen *et al*. 2006; Timling *et al*. 2012), Alaska, USA (Walker *et al*. 2011; Timling *et al*. 2012) and Antarctic Peninsula (Gonçalves *et al*. 2015; Cid *et al*. 2017; Martorell *et al*. 2017; Ferreira *et al*. 2019; Molina–Montenegro *et al*. 2019; Rosa *et al*. 2009, 2010; Teixeira *et al*. 2010; Santiago *et al*. 2012; da Silva *et al*. 2017; Santiago, Rosa and Rosa 2017; Wentzel *et al*. 2019; Silva *et al*. 2020; Zhang *et al*. 2020). Pie charts show averages of the relative abundances of the major bacterial and fungal phyla analysed by culturable-independent approaches from alpine (Jorquera *et al*. 2016; Lu *et al*. 2016; Kumar *et al*. 2017; Li *et al*. 2018; Roy *et al*. 2018; Praeg, Pauli and Illmer 2019; Wassermann *et al*. 2019; Jamil *et al*. 2020), Arctic (Walker *et al*. 2011; Nissinen, Männistö and van Elsas 2012; Blaalid *et al*. 2014; Zhang and Yao 2015; Mundra, Bahram and Eidesen 2016; Kumar *et al*. 2017; Lorberau *et al*. 2017; Given *et al*. 2020) and Antarctic (Jorquera *et al*. 2016; Molina–Montenegro *et al*. 2019; Teixeira *et al*. 2010; Silva *et al*. 2020; Zhang *et al*. 2020) regions. It should be noted that these studies used various primers, PCR conditions and sequencing platforms. No culturable-independent studies on plant-associated fungal communities in Antarctic regions are available till now.

The taxonomic structure of plant-associated bacterial and fungal communities was analysed by culture-independent approaches in alpine regions (Fig. 1 and Table 1), such as European Alps (Garnica *et al*. 2013; Casazza *et al*. 2017; Roy *et al*. 2018; Praeg, Pauli and Illmer 2019; Wassermann *et al*. 2019), Hindu Kush, Karakorum–Himalaya and Tibetan Plateau (Pan *et al*. 2013; Li *et al*. 2014, 2018; Řeháková *et al*. 2015; Angel *et al*. 2016; Lu *et al*. 2016; Kotilínek *et al*. 2017; Chang *et al*. 2018; Jamil *et al*. 2020), Andes (Correa-Galeote *et al*. 2016; Jorquera *et al*. 2016; Ruiz-Pérez, Restrepo and Zambrano 2016; Senés-Guerrero and Schüssler 2016; Pfeiffer *et al*. 2017; Chica *et al*. 2019) and Rocky Mountains (Schmidt *et al*. 2008; Bueno de Mesquita *et al*. 2018). Proteobacteria, Actinobacteria, Acidobacteria and Bacteroidetes were consistently found as dominant taxa in the rhizosphere and plant tissues of the analysed plants (e.g. *Arenaria*, *Astrantia, Blechnum, Chionocharis, Draba*, *Espeletia*, *Euphrasia, Equisetum, Gentiana, Gentianella, Ladakiella*, *Miscanthus*, *Oxalis, Poa*, *Ranunculus, Saussurea, Tropaeolum, Ullucus* and *Waldheimia* genera; Řeháková *et al*. 2015; Angel *et al*. 2016; Correa-Galeote *et al*. 2016; Jorquera *et al*. 2016; Ruiz-Pérez, Restrepo and Zambrano 2016; Pfeiffer *et al*. 2017; Chang *et al*. 2018; Chica *et al*. 2019; Praeg, Pauli and Illmer 2019; Wassermann *et al*. 2019; Huang *et al*. 2020). Other bacterial phyla, such as Planctomycetes, Chloroflexi, *Candidatus* Dormibacteraeota (formerly AD3), Verrucomicrobia, Gemmatimonadetes and Firmicutes and were less frequently found in the plant species mentioned above (Correa-Galeote *et al*. 2016; Jorquera *et al*. 2016; Ruiz-Pérez, Restrepo and Zambrano 2016; Pfeiffer *et al*. 2017; Chang *et al*. 2018; Chica *et al*. 2019; Praeg, Pauli and Illmer 2019; Huang *et al*. 2020). At low taxonomic level, dominant bacterial genera of plant-associated communities in alpine regions were *Candidatus Solibacter* and *Rhodoplanes* in *Arenaria polytrichoides* and *Chionocharis hookeri* (Chang *et al*. 2018) or *Streptomyces*, *Arthrobacter* and *Paenibacillus* in *Thylacospermum caespitosum* (Řeháková *et al*. 2015), indicating high taxonomic complexity of bacterial communities associated to alpine plants. Among the plant-associated Archaea, Crenarchaeota and Thaumarchaeota were the most abundant phyla in plant seeds, rhizosphere and roots in alpine regions (Ruiz-Pérez, Restrepo and Zambrano 2016; Praeg, Pauli and Illmer 2019; Wassermann *et al*. 2019).

**Table 1.** Summary of culture-independent studies on the structural diversity of plant-associated microbial communities in cold regions.

Cold region	Geographical location	Altitude (m)	Plants and tissues analysed	Microbial parameters and methodology	Main findings	Reference
Alpine	Central Alps of Tyrol (Austria)	2600–3400	*Ranunculus glacialis;* rhizosphere and bulk soil	Bacteria, archaea and fungi; Illumina MiSeq	Planctomycetales, Actinomycetales, Rhizobiales, Spartobacteria unclassified, Burkholderiales, Sphingobacteriales and Rhodospirillales were the most abundant bacterial orders, whereas Helotiales, Mortierellales, Pleosporales, Dothideomycetes order incertae sedis, Sporidiobolales, Hypocreales, Chaetothyriales and Lecanorales were the most abundant fungal orders. *Nitrososphaera* spp. were the dominant taxa among archaeal communities. Altitude and temperature were the main factors influencing community structure	Praeg, Pauli and Illmer (2019)
	Northern Calcareous Alps, Hochschwab region (Austria)	NA	*Astrantia major*, *Euphrasia rostkoviana*, *Gentiana asclepiadea*, *Gentianella germanica*, *Heliosperma quadrifida*, *Parnassia palustris*, *Rhinanthus glacialis* and *Scabiosa lucida*; seeds	Bacteria, archaea and fungi; Illumina MiSeq	Bacteria and fungi were abundant while archaea were less abundant in the seeds. *Sphingomonas*, *Pseudomonas*, *Tatumella*, *Pantoea*, Soil Crenarchaeotic Group, *Cryptococcus*, *Cladosporium* and *Davidiella* were the highly abundant genera shared between seed core microbiome. Plant genotype (species) was the main factor affecting community composition	Wassermann *et al*. (2019)
	Baima Snow Mountain, Yunnan Province (China)	4780	*A. polytrichoides* and *C. hookeri*; rhizosphere and bulk soil	Bacteria; Illumina HiSeq	*Candidatus Solibacter*, *Mycobacterium* and *Rhodoplanes* were among the most abundant genera. Plant species and soil sulfur content were the major factors affecting the community structure	Chang *et al*. (2018)
	Mount Shukule II, Tibetan Plateau, Ladakh (India)	6150	*Draba alshehbazii*, *Draba altaica*, *Ladakiella klimesii*, *Poa attenuata*, *Saussurea gnaphalodes* and *W. tridactylites*; roots and bulk soil	Bacteria; Illumina MiSeq	Sphingomonadales (Proteobacteria phylum) and Sphingobacteriales (Bacteroidetes phylum) were the dominant orders	Angel *et al*. (2016)
	North West Himalaya, Ladakh (India)	4850–5850	*T. ceaspitosum*; rhizosphere and bulk soil	Bacteria; Single Strand Conformation Polymorphism and culture-dependent analysis	Actinobacteria dominated the cultivable communities and *Streptomyces*, *Arthrobacter* and *Paenibacillus* were the most abundant genera. Soil texture was the most important factor for the community structure and bacterial count	Řeháková *et al*. (2015)
	Andean highland (Ecuador)	3700	*Oxalis tuberosa*, *Tropaeolum tuberosum* and *Ullucus tuberosus*; rhizosphere and bulk soil	Bacteria; Illumina MiSeq	*Opitutaceae*, *Methilophilaceae*, *Sphingobacteraceae*, *Chitinophagaceae*, *Flavobacteraceae*, *Sphingomonadaceae* and *Burkholderaceae* were the most abundant families in all plants	Chica *et al*. (2019)
	Andes, Quechua region (Peru)	3537	*Zea mays*; rhizosphere and bulk soil	Bacteria; 454 pyrosequencing	Gp6 and *Rhodoferax* were the most abundant genera in the rhizosphere	Correa-Galeote *et al*. (2016)
	Andes, Huancavelic, Sincos-Junin and Sicaya-Junin regions (Peru)	3245–4070	*S. tuberosum*; rhizosphere	Bacteria; 454 pyrosequencing	Three rhizosphere microbiome components were proposed; opportunistic microbiome comprised of occasionally occurring or specifically enriched OTUs, stable core microbiome (*Bradyrhizobium*, *Sphingobium*, *Microvirga*, *Blastococcusi* and *SMB53*) continuously abundant in all samples and vegetation stages and dynamic core microbiome comprised of OTUs enriched at specific vegetation stages	Pfeiffer *et al*. (2017)
	Andes, Natural National Park Los Nevados (Columbia)	NA	*Espeletia* sp.; leaves (young and matured), necromass (senescent leaves) and roots	Bacteria and archaea; Illumina MiSeq	*Acinetobacter*, *Candidatus Baumannia*, *Burkholderia*, *Erwinia*, *Hymenobacter*, *Klebsiella*, *Pseudomonas*, *Propionibacterium* and *Sphingomonas* were the most common genera shared by all plant tissues	Ruiz-Pérez, Restrepo and Zambrano (2016)
	North West Himalaya, Ladakh (India)	3400–6150	62 host species including *Leontopodium ochroleucum*, *P. attenuata*, *Potentilla multifida*, *Saxifraga cernua*, *Saxifraga nanella*, *Stellaria decumbens* and *Tanacetum pyrethroides*; roots	AMF and DSE; microscopy and Roche sequencing	The highest diversity and abundance of AMF communities along the elevational gradient in the dry Himalayas were found in the moderately stressful mesic steppes rather than in extreme environments	Kotilínek *et al*. (2017)
	Andes (Bolivia, Ecuador and Peru)	2658–4075	*S. tuberosum*; roots	AMF; 454 pyrosequencing	*Acaulospora* spp. were identified as dominant colonizers, co-occurring with *Cetraspora nodosa* and certain *Claroideoglomus* and *Rhizophagus* species in most potato root samples	Senés-Guerrero and Schüssler (2016)
	Gangcha steppe, Qinghai Province (China)	3265	*S. purpurea*; rhizosphere and roots	Fungi; cloning and Sanger sequencing	Chaetothyriales, Eurotiales, Acarosporales and Mortierellales were the dominant orders in the rhizosphere, whereas Agaricales, Sordariales, Helotiales, Mitosporic Ascomycota and Hypocreales were the dominant orders in the roots	Lu *et al*. (2016)
	French Hautes‐Alpes (France)	2100–3050	*Silene acaulis*; rhizosphere and bulk soil	Fungi; Illumina MiSeq	*Cladosporiaceae and Dermateaceae* families (Ascomycota phylum) were the dominant taxa in the rhizosphere. Bedrock and plant genotype influence fungal recruitment	Roy *et al*. (2018)
	Yunnan Province (China)	3260	*D. indica*; rhizosphere	Fungi; Illumina MiSeq	*Mortierella*, *Gibberella*, *Cilliophora*, *Zopifiella*, unclassified_p_Ascomycota and unclassified_o_Pleosporales were the dominant taxa at the three altitudes investigated. Fungal diversity increases across the altitude gradient	Jamil *et al*. (2020)
	Gansu and Inner Mongolia provinces (China)	533–3075	*S. krylovii*; roots	Fungi; Illumina MiSeq	*Marasmius*, *Fusarium*, *Acremonium*, *Sarcinomyces* and *Monosporascus* were the dominant genera	Li *et al*. (2018)
	Bavarian Alps (Germany)	1020–1830	70 host species including *A. major*, *Bistorta vivipara*, *Campanula scheuchzeri*, *Daphne striata*, *Globularia nudicaulis*, *Lamium* cf*. montanum*, *Pinguiculaalpina*, *Poaceae* sp., *Polygala* cf*. alpestris*, *Soldanella alpina*, *Trifolium badium*, *Trifolium pratense*, *Trifolium repens*, *Urtica dioica*, *Vaccinium myrtillus* and *Viola reichenbachiana*; roots	Sebacinales communities; cloning and Sanger sequencing	Sebacinales appear to occur in low abundance but they are phylogenetically diverse and widespread in the ecosystems studied (montane and subalpine). Land use, pH and humus content influenced the diversity and assembly of Sebacinales communities	Garnica *et al*. (2013)
	Mount Segrila, Tibetan Plateau, Tibet (China)	3446–4556	*Kobresia* sp. and *P. centrasiaticum*; roots	AMF; cloning and Sanger sequencing	*Acaulosporaceae* and *Glomeraceae* were the dominant families. Elevation, plant species and soil variables were the most significant factors affecting the AMF community across all elevations	Li *et al*. (2014)
	Zhadang Glacier, Tibetan Plateau, Tibet (China)	5500	*Melandrium apetalum* and *Poa litwinowiana*; roots and rhizosphere	AMF and DSE; microscopy, cloning and Sanger sequencing	Both AMF and DSE fungi synchronously colonized the two plant species, but AMF dominated in *M. apetalum* and DSE dominated in *P. litwinowiana*	Pan *et al*. (2013)
	Rocky Mountains, Colorado (USA)	3636–3933	35 host species including *Besseya alpina*, *Carex* spp., *S. acaulis*, *Deschampsia cespitosa*, *Geum rossii*, *Oreoxis alpina*, *Oxyria digyna* and *Senecio fremontii*; roots	AMF and DSE; microscopy and Illumina MiSeq	AMF were more abundant in roots at lower elevation areas with lower snowpack and lower phosphorus and nitrogen content, whereas DSE colonization was highest in areas with less snowpack and higher inorganic nitrogen levels. *Acaulospora*, *Entrophospora*, *Archaeospora*, *Claroideoglomus* and *Glomus* were the most widespread AMF genera, whereas *Phialophora*, *Capronia*, *Leptosphaeria*, *Exophiala* and *Cryptosporiopsis* were the most widespread DSE genera	Bueno de Mesquita *et al*. (2018)
	Rocky Mountains, Colorado (USA) and Andes (Peru)	4298–5391	18–30 host species including *Artemisis* spp., *Astragalus* cf*. arequipensis*, *Draba* spp., *Perezia coerulescens*, *Polemonium* spp., *Trifolium* spp., *Valerianapycnantha*, *Werneria orbignyana* and *Xenophyllum rosenii*; roots	AMF and DSE; microscopy	AMF were absent in the two species of plants sampled (both *Compositae*) but roots of both were heavily colonized by DSE fungi at the highest sites in the Andes (5391 m). AMF were present in roots while DSE fungi were rare in plants outside of *Compositae* at slightly lower elevations (5240–5250 m). AMF were present, but at very low levels and all plants sampled contained DSE fungi at the highest sites sampled in Colorado	Schmidt *et al*. (2008)
	South-western Alps (Italy and France)	2039–2408	*B. subacaulis*; roots	AMF; microscopy, cloning and Sanger sequencing	*Glomeraceae* was the dominant family. Soil quality and slope influenced the AMF diversity	Casazza *et al*. (2017)
Artic	Kilpisjärvi fell area (Finland)	559–898	*Diapensia lapponica*, *Juncus trifidus* and *O. digyna*; whole plant and seeds	Bacteria; cloning and Sanger sequencing	*Sphingomonas* spp. were characteristic for *D. lapponica* and *O. digyna*. Plant species and snow cover affected the community compositions	Nissinen, Männistö and van Elsas (2012)
	Kilpisjärvi fell area (Finland)	925	*O. digyna;* leaves and roots	Bacteria; Ion Torrent sequencing	Firmicutes was highly abundant in the leaf communities of bait and wild plants. Proteobacteria and Bacteroidetes were more abundant in the roots, albeit with different relative abundances in bait and wild plant groups. Tissue type and plant group had strong impact on the community structure	Given *et al*. (2020)
	Svalbard archipelago (Norway)	NA	*B. vivipara*; roots	Fungi; 454 pyrosequencing	Basidiomycota and Ascomycota (particularly Thelephorales, Agaricales, Pezizales and Sebacinales orders) were the dominant taxa	Blaalid *et al*. (2014)
	Svalbard archipelago (Norway)	NA	*Cassiope tetragona*; roots	Fungi; Illumina Miseq	Sebacinales and Agaricales orders (Basidiomycota phylum), particularly *Clavaria*, *Cortinarius* and *Mycena* genera, were the dominant taxa	Lorberau *et al*. (2017)
	Svalbard archipelago (Norway)	10–67	*C. tetragona*, *Saxifraga cespitosa*, *Saxifraga oppositifolia* and *S. acaulis*; roots	Fungi, 454 pyrosequencing	Helotiales, Pleosporales, Capnodiales and Tremellales orders (particularly *Cryptococcus*, *Rhizosphaera*, *Mycopappus*, *Melampsora*, *Tetracladium*, *Phaeosphaeria*, *Mrakia*, *Venturia* and *Leptosphaeria* genera) were the dominant taxa	Zhang and Yao (2015)
	Svalbard archipelago (Norway)	55	*B. vivipara*; roots	Fungi; Illumina Miseq	Stress-tolerant genera such as *Laccaria* and *Hebeloma* were abundant in nutrient-poor soil whereas functional competitors genera such as *Lactarius* and *Russula* were dominant in the nutrient-rich soil	Mundra, Bahram and Eidesen (2016)
	Svalbard archipelago (Norway)	NA	*B. vivipara*, *Dryas octopetala* and *Salix polaris*; roots	Fungi, 454 pyrosequencing	No evidence of host specificity and no significant differences in fungal OTU richness were observed across the three plant species	Botnen *et al*. (2014)
	Svalbard archipelago (Norway)	NA	31 host species including *Carex rupestris*, *Luzula confuse*, *Micrantes nivalis*, *O. digyna*, *Papaver dahliana*, *Potentilla puchella*, *Ranunculus* sp., *Taraxacum arcticum* and *Trisetum spicatum*; roots	Fungi; Illumina Miseq and HiSeq	Helotiales, Pleosporales, Chaetothyriales and Sordariales were the dominant orders in most of the plants. Plant species and to a less extent environmental factors affected the community structure	Botnen *et al*. (2020)
	Svalbard archipelago (Norway)	NA	13 host species including *Alopecurus ovatus*, *Coptidium spitsbergense*, *Deschampsia alpina*, *Festuca rubra* ssp. *richardsonii*, *Poa* spp., *Ranunculus* spp., *T. arcticum* and *T. spicatum*; roots	AMF; microscopy	No associations between the abundances of AMF structures in roots and edaphic factors (pH, soil moisture, carbon, nitrogen and phosphorus concentrations and total organic matter)	Newsham *et al*. (2017)
	Kilpisjärvi fell area (Finland)	600	*Avenella flexuosa*; roots	AMF and DSE; microscopy	AMF colonization was high at open coastal dunes, whereas DSE fungi were more common at forested sites, in the boreal zone. Humus thickness affected AMF fungi negatively and DSE fungi positively	Kauppinen *et al*. (2014)
	Saskatoon and Axel Heiberg Island (Canada)	NA	*Arnica alpina*, *Epilobium latifolium*, *Erigeron* spp., *Ranunculus nivalis* and *Taraxacum* spp.; roots	AMF; microscopy	AMF colonization exceeded 80% for Arctic Asteraceae, similar to 66–90% for prairie *Taraxacum* and *Erigeron*. Soil depth did not influence AMF colonization	Allen *et al*. (2006)
	Canadian Arctic Archipelago (Canada); Alaska (USA) and Greenland (Denmark)	NA	*Dryas integrifolia* and *Salix arctica*; roots	Ectomycorrhizal fungi; microscopy, cloning and Sanger sequencing	*Thelephoraceae*, *Inocybaceae*, *Sebacinaceae*, *Cortinariaceae* and *Pyronemataceae* were the dominant families. Environmental factors corresponding to glaciation history, geology, soil properties, plant productivity and climate were the main factors affecting community structure	Timling *et al*. (2012)
	Alaska (USA)	726–752	*C. tetragona*, *Empetrum nigrum* and *Vaccinium vitis-idaea*; roots	Fungi; cloning, Sanger sequencing and culture-dependent analysis	Helotiales was the dominant order. *Rhizoscyphus ericae* complex and *Phialocephala–Acephala* complex dominated the communities analysed by cloning and sequencing and culture-dependent approaches, respectively	Walker *et al*. (2011)
Antarctica	Devils Point, Livingstone Island (Antarctic peninsula)	NA	*C. quitensis* and *D. antarctica*; rhizosphere	Bacteria; Illumina MiSeq	Proteobacteria, Actinobacteria, Bacteroidetes, Acidobacteria and Verrucomicrobia were the most abundant phyla	Molina-Montenegro *et al*. (2019)
	King George Island (Antarctic peninsula)	NA	*D. antarctica*; rhizosphere	Bacteria; Ion Torrent PGM and culture-dependent analysis	Actinomycetales (Actinobacteria phylum) was the dominant order. *Actinoplanes*, *Arthrobacter*, *Kribbella*, *Mycobacterium*, *Nocardia*, *Pilimelia*, *Pseudarthrobacter*, *Rhodococcus*, *Streptacidiphilus*, *Streptomyces* and *Tsukamurella* genera belonging to Actinobacteria were isolated	Silva *et al*. (2020)
	King George Island (Antarctic peninsula)	NA	*C. quitensis* and *D. antarctica*; rhizosphere	Bacteria; 454 pyrosequencing and denaturing gradient gel electrophoresis	Firmicutes was the most abundant phylum in most samples, and there were high levels of anaerobic representatives	Teixeira *et al*. (2010)
	Deception Island (Antarctic peninsula)	NA	*C. quitensis* and *D. antarctica*; rhizosphere, leaves, roots	Bacteria; Illumina MiSeq	Co-occurrences network analyses identified putative niche-specific keystone taxa. In particular, *Microbacteriaceae*, *Pseudomonaceae*, *Lactobacillaceae* and *Corynebacteriaceae* in the endosphere; *Chitinophagaceae* and *Sphingomonadaceae* in the phyllosphere, and *Rhodospirillaceae* in the rhizosphere	Zhang *et al*. (2020)
Alpine and Arctic	Alps, Mayrhofen (Austria); Kilpisjärvi (Finland) and Ny-Ålesund (Norway)	2400	*O. digyna* and *S. oppositifolia*; rhizosphere, roots and bulk soil	Bacteria; Ion Torrent sequencing	Relative abundances of Proteobacteria decreased progressively from the alpine to the Arctic, whereas those of Actinobacteria increased. Firmicutes, Proteobacteria and Bacteroidetes dominated the endosphere communities. Plant compartments impacted bacterial diversity and community structures more than geographic region or sampling site	Kumar *et al*. (2017)
	Tromsø, Finse and Svalbard archipelago (Norway)	20–1480	*D. octopetala*; roots	Fungi; cloning and Sanger sequencing	*Cenococcum*, *Cortinarius*, *Hebeloma*, *Inocybe* and *Tomentella* were the most occurred genera. Fungal diversity does not decrease in high latitude arctic regions	Bjorbækmo *et al*. (2010)
Alpine and Antarctica	Andes (Chile)	NA	*Blechnum chilense* and *Equisetum arvense*; rhizosphere	Bacteria; 454 pyrosequencing and denaturing gradient gel electrophoresis	Alphaproteobacteria and *Burkholderia* were the dominant class and genus, respectively, in both regions	Jorquera *et al*. (2016)
	King George Island (Antarctic peninsula)		*C. quitensis* and *D. antarctica*; rhizosphere			

Abbreviations: NA = not available; AMF = arbuscular mycorrhizal fungi and DSE = dark septate endophytes.

In the majority of the studies, Ascomycota and Basidiomycota were found as dominant phyla among plant-associated fungi, followed by Glomeromycota, Mucoromycota and Zygomycota, in the rhizosphere and roots of several plants in alpine regions, such as *Dryas*, *Duchesnea*, *Empetrum*, *Polemonium*, *Ranunculus*, *Salix*, *Silene*, *Stipa*, *Taraxacum* and *Vaccinium* species (Ryberg, Larsson and Molau 2009; Lu *et al*. 2016; Toju, Tanabe and Ishii 2016; Li *et al*. 2018; Roy *et al*. 2018; Praeg, Pauli and Illmer 2019; Jamil *et al*. 2020). The dominant fungal genera were *Psathyrella*, *Armillaria*, *Sordariales*, *Helotiales* and *Cylindrocarpon* in *Stipa purpurea* (Lu *et al*. 2016) and *Marasmius*, *Fusarium*, *Acremonium*, *Sarcinomyces* and *Monosporascus* in *Stipa krylovii* (Li *et al*. 2018). In the alpine plant *Duchesnea indica*, various genera (e.g. *Mortierella*, *Gibberella*, *Cilliophora* and *Zopifiella*) were consistently found across three different altitude gradients (Jamil *et al*. 2020). Furthermore, ectomycorrhizal communities in alpine regions were dominated by *Cenococcum*, *Thelephoraceae* and *Cortinarius* genera (Ryberg, Larsson and Molau 2009). Among mycorrhizal fungi, Glomeraceae (*Glomus* spp.), Acaulosporaceae (*Acaulospora* spp.and *Entrophospora* spp.) and Diversisporaceae (*Diversispora* spp.) were found as dominant in a wide variety of alpine plant species, such as *Astragalus polycladus*, *Berardia subacaulis*, *Dracocephalum heterophyllum*, *Kobresia* sp., *Leontopodium nanum*, *Pennisetum centrasiaticum*, *Poa attenuate*, *Polemonium viscosum*, *Potentilla bifurca*, *St. krylovii, St. purpurea*, *Solanum tuberosum*, *Taraxacum ceratophorum*, *Taraxacum officinale* and *Waldheimia tridactylites* (Liu *et al*. 2011, 2015; Becklin, Hertweck and Jumpponen 2012; Li *et al*. 2014; Senés-Guerrero and Schüssler 2016; Zhang *et al*. 2016; Casazza *et al*. 2017; Kotilínek *et al*. 2017; Bueno de Mesquita *et al*. 2018; Haug, Setaro and Suárez 2019). Other important fungal groups studied in alpine regions include dark septate endophytes (DSE) and Sebacinales, belonging to the Ascomycota and Basidiomycota phylum, respectively (Schmidt *et al*. 2008; Urcelay, Acho and Joffre 2011; Garnica *et al*. 2013; Pan *et al*. 2013; Bueno de Mesquita *et al*. 2018). DSE genera (e.g. *Phialophora*, *Capronia*, *Leptosphaeria*, *Exophiala* and *Cryptosporiopsis*) were widespread among plant roots (e.g. forbes, grasses and sedges) in the Rocky Mountains (Bueno de Mesquita *et al*. 2018). In contrast, low abundance of Sebacinales communities were found in roots of 70 plant species of the Bavarian Alps (Garnica *et al*. 2013).

A limited number of studies investigated the taxonomic structure of both plant-associated prokaryotes and fungi (Praeg, Pauli and Illmer 2019; Wassermann *et al*. 2019). In particular, the relative abundance of bacterial (74.9%) and fungal (24.9%) communities was higher compared to that of archaeal communities (0.05%) in seeds of eight plant species in Alpine meadows (Austria), and the dominant phyla were Proteobacteria, Actinobacteria, Bacteroidetes, Ascomycota, Basidiomycota and Thaumarchaeota (Wassermann *et al*. 2019). Furthermore, Praeg, Pauli and Illmer (2019) identified dominant bacterial (e.g. Actinomycetales, Burkholderiales, Rhizobiales, Sphingobacteriales, Sphingomonadales, and Xanthomonadales) archaeal (e.g. *Nitrososphaera* spp.) and fungal (e.g. Cystofilobasidiales, Helotiales, Mortierellales and Tremellales) taxa in the rhizosphere of *Ranunculus glacialis* at different altitudinal zones.

Culturable plant-associated bacteria and fungi have been investigated in Alpine regions (Calvo *et al*. 2010; Sheng *et al*. 2011; Bisht, Mishra and Joshi 2013; Cui *et al*. 2015; Řeháková *et al*. 2015; Castellano-Hinojosa *et al*. 2018; Oberhofer *et al*. 2019; Chumpitaz-Segovia *et al*. 2020; Ma *et al*. 2020; Tapia-Zubek *et al*. 2009; Wang *et al*. 2016; Tapia-Vázquez *et al*. 2020; Ulloa-Muñoz *et al*. 2020). In particular, Oberhofer *et al*. (2019) obtained 77 actinobacterial isolates belonging to *Actinokineospora, Kitasatospora, Asanoa, Microbacterium, Micromonospora, Micrococcus, Mycobacterium, Nocardia* and *Streptomyces* genera in the rhizosphere of an alpine medicinal plant (*Leontopodium nivale* subsp. *alpinum*). Likewise, Actinobacteria (*Streptomyces* and *Arthrobacter*) dominated the culturable fraction of bacterial communities in the rhizosphere of *T. caespitosum* in the Himalayas (Řeháková *et al*. 2015). In the Tibetan Plateau, 50 endophytic bacterial isolates were obtained from various tissues of *Kobreasia capillifolia* and *Bacillus* was the dominant genus (Wang *et al*. 2016). Moreover, Ulloa-Muñoz and colleagues (2020) investigated the taxonomic structure of endophytic plant-growth promoting microorganisms from two wild medicinal plants (*Gentianella weberbaueri* and *Valeriana pycnantha*) in the Peruvian Andes and isolated six endophytic fungi belonging to *Pyrenochaeta*, *Scleroconidioma*, *Cryptococcus* and *Plenodomus* genera. Overall, taxonomic evidences obtained from these studies suggested that plants growing in alpine regions harbor rare and underexplored microbial species, which can be targeted for isolation and functional characterization in the future.

**Figure 2.**
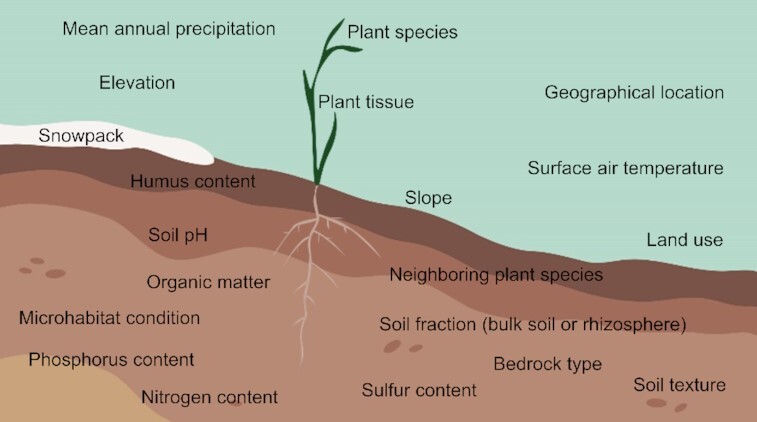
Main factors affecting the taxonomic structure of plant-associated microbial communities in cold environments. Factors affecting the taxonomic structure of plant-associated microbial communities are summarized according to the literature (Becklin, Hertweck and Jumpponen 2012; Blaalid *et al*. 2014; Botnen *et al*. 2019; Abrego *et al*. 2020; Arraiano-Castilho *et al*. 2020; Fujimura and Egger 2012; Garnica *et al*. 2013; Kauppinen *et al*. 2014; Ciccazzo *et al*. 2016; Kumar *et al*. 2016; Casazza *et al*. 2017; Koizumi and Nara 2017; Kotilínek *et al*. 2017; Bueno de Mesquita *et al*. 2018; Chang *et al*. 2018; Given *et al*. 2020, 2017; Li *et al*. 2014, 2018; Tscherko *et al*. 2005; Upson, Newsham and Read 2008; Nissinen, Männistö and van Elsas 2012; Timling *et al*. 2012; Welc *et al*. 2014; Massaccesi *et al*. 2015; Mundra *et al*. 2015; Řeháková *et al*. 2015; Mundra, Bahram and Eidesen 2016; Yang *et al*. 2016; Zhang *et al*. 2016; Lorberau *et al*. 2017; Santiago, Rosa and Rosa 2017; Mapelli *et al*. 2018; Roy *et al*. 2018; Wassermann *et al*. 2019, 2020; Zhang and Yao 2015).

In Alpine regions, the plant species was one of the main factors shaping the plant-associated bacteria (Massaccesi *et al*. 2015; Chang *et al*. 2018; Wassermann *et al*. 2019) and fungi (Tscherko *et al*. 2005; Becklin, Hertweck and Jumpponen 2012; Li *et al*. 2014; Welc *et al*. 2014; Massaccesi *et al*. 2015; Bueno de Mesquita *et al*. 2018; Roy *et al*. 2018), indicating host specificity of plant-associated communities (Fig. 2). Environmental-related factors can also influence the taxonomic structure of plant-associated microbial communities in alpine regions, such as mean annual precipitation (Zhang *et al*. 2016; Li *et al*. 2018), elevation and snowpack (Yang *et al*. 2016; Kotilínek *et al*. 2017; Bueno de Mesquita *et al*. 2018), microhabitat condition (Koizumi and Nara 2017), soil fraction (bulk soil or rhizosphere; Massaccesi *et al*. 2015), soil texture (Řeháková *et al*. 2015), soil sulfur content (Chang *et al*. 2018), soil pH and total nitrogen (Bueno de Mesquita *et al*. 2018; Arraiano-Castilho *et al*. 2020), slope (Casazza *et al*. 2017), land use and humus content (Garnica *et al*. 2013). Unfortunately, the plant microbiota was rarely studied in some alpine regions, such as the tropical Afroalpine mountain, Southern Alps (New Zealand), Urals, Caucasus and Pamir, indicating that further investigations on those less studied areas is required for a comprehensive understanding of the effect of the location on the taxonomic structure of plant-associated microbial communities (Ciccazzo *et al*. 2016). Likewise, studies on the variability of the taxonomic structure of endophytic microbial communities that considers different plant tissues in various plant species across various location may clarify the tissue-specificity in alpine plants.

## PLANT-ASSOCIATED MICROBIAL COMMUNITIES IN ARCTIC REGIONS

The taxonomic structure of plant-associated bacterial and fungal communities was analysed in European Arctic (e.g. Lapland, Finland and Svalbard, Norway) and in North American Arctic (e.g. northern Canada and Alaska) regions (Fig. 1 and Table 1) by culture-independent approaches (Allen *et al*. 2006; Bjorbækmo *et al*. 2010; Walker *et al*. 2011; Nissinen, Männistö and van Elsas 2012; Timling *et al*. 2012; Blaalid *et al*. 2014; Botnen *et al*. 2014, 2020; Kauppinen *et al*. 2014; Zhang and Yao 2015; Mundra, Bahram and Eidesen 2016; Kumar *et al*. 2017; Lorberau *et al*. 2017; Newsham *et al*. 2017; Given *et al*. 2020). Proteobacteria, Actinobacteria, Acidobacteria, *Candidatus* Dormibacteraeota (formerly AD3), Bacteroidetes and Firmicutes were found as dominant bacterial phyla in various plant compartments (e.g. rhizosphere, root endosphere and phyllosphere) of *Diapensia lapponica*, *Juncus trifidus*, *Oxyria digyna* and *Saxifraga oppositifolia* (Nissinen, Männistö and van Elsas 2012; Kumar *et al*. 2017; Given *et al*. 2020). However, the diversity of endophytic bacterial communities was lower in leaf compared to root tissues of *O. digyna*, with Proteobacteria and Bacteroidetes that dominated root tissues and Firmicutes that dominated leaf tissues (Given *et al*. 2020), confirming plant tissue specificity of bacterial communities also in Arctic environments.

Among fungal communities, Basidiomycota was the dominant phylum in roots of several Arctic plants, such as *Bistorta* spp., *Cassiope* spp., *Dryas* spp. and *Salix* spp. (Bjorbækmo *et al*. 2010; Timling *et al*. 2012; Blaalid *et al*. 2014; Botnen *et al*. 2014; Mundra, Bahram and Eidesen 2016; Lorberau *et al*. 2017). Conversely, Ascomycota was reported as the dominant phylum in Ericaceae species (e.g. *Cassiope*, *Empetrum* and *Vaccinium*) and non-mycorrhizal plant species (e.g. *Deschampsia*, *Draba*, *Carex*, *Luzula*, *Pedicularis*, *Ranunculus*, *Silene* and *Saxifraga*; Walker *et al*. 2011; Zhang and Yao 2015; Botnen *et al*. 2020), indicating host specificity of plant-associated fungal communities in Arctic environments. In particular, *Cryptococcus*, *Rhizosphaera*, *Mycopappus*, *Melampsora*, *Tetracladium*, *Phaeosphaeria*, *Mrakia*, *Venturia* and *Leptosphaeria* genera were consistently detected in association with Arctic plants (Zhang and Yao 2015). Moreover, ectomycorrhizal fungal communities were dominated by *Thelephora*, *Tomentella*, *Sebacina*, *Inocybe*, *Cortinarius*, *Russula*, *Hebeloma*, *Laccaria*, *Clavulina* genera in the North American Arctic samples (Timling *et al*. 2012) and by *Cenococcum*, *Cortinarius*, *Hebeloma*, *Inocybe* and *Tomentella* genera in the European Arctic samples (Bjorbækmo *et al*. 2010). Likewise, dominant fungal genera associated with *Bistorta vivipara* roots were *Laccaria* and *Hebeloma* in a nutrient-poor soil, and *Lactarius* and *Russula* in a nutrient-rich soil (Mundra, Bahram and Eidesen 2016), indicating environmental niche differentiation of plant-associated fungal communities. However, mycorrhizal fungi (e.g. *Glomus* spp., *Archaeospora* spp. and *Claroideoglomus* spp.) and DSE (e.g. *Cadophora* spp. and *Phialocephala* spp.) fungi were found as low abundant taxa in Arctic plants (Allen *et al*. 2006; Bjorbaekmo *et al*. 2010; Öpik *et al*. 2013; Kauppinen *et al*. 2014; Newsham *et al*. 2017; Botnen *et al*. 2020).

Culturable plant-associated bacteria and fungi have been characterized in Arctic regions (Higgins *et al*. 2007; Walker *et al*. 2011; Nissinen, Männistö and van Elsas 2012; Poosakkannu, Nissinen and Kytöviita 2015). From three different host plants, Nissinen, Männistö and van Elsas (2012) obtained a collection of 325 endophytic bacterial isolates belonging to 56 genera of five phyla (Actinobacteria, Bacteroidetes, Firmicutes, Acidobacteria and Proteobacteria), with members of *Burkholderia* spp. and *Sphingomonas* spp. being the most abundant. Culturable endophytic microorganisms (178 bacterial and 30 fungal isolates) were also obtained from various plant tissues (leaf, root, seed and seedling) of *Deschampsia flexuosa* and specific taxa were isolated according to the plant tissue (Poosakkannu, Nissinen and Kytöviita 2015). For example, isolates closely related to *Burkholderia sordidicola* were present in leaf and root samples of both successional stages (sand and forest), while isolates closely related to *Curtobacterium flaccumfaciens* were present only in the leaf and root samples from the sand (Poosakkannu, Nissinen and Kytöviita 2015).

In Arctic regions, host-related factors (e.g. plant species and tissue type) and environmental-related factors (e.g. geographic location) affected the taxonomic structure of endophytic and rhizospheric bacterial communities (Nissinen, Männistö and van Elsas 2012; Kumar *et al*. 2016, 2017; Mapelli *et al*. 2018; Given *et al*. 2020; Fig. 2), indicating host-specific adaptations and environmental niche differentiation of bacterial communities. Likewise, host plants, neighboring plants (Mundra *et al*. 2015; Lorberau *et al*. 2017; Abrego *et al*. 2020; Botnen *et al*. 2020) and environmental factors (e.g. mean annual temperature and precipitation, elevation, humus content, organic matter, bedrock type, phosphorus and nitrogen content and soil pH) can affect the taxonomic structure of fungal communities associated with Arctic plants (Fujimura and Egger 2012; Timling *et al*. 2012; Blaalid *et al*. 2014; Kauppinen *et al*. 2014; Mundra, Bahram and Eidesen 2016; Botnen *et al*. 2019; Abrego *et al*. 2020). However, plant species have variable effect on the plant-associated fungal communities depending on the fungal group (e.g. effect on root associated-endophytes and but no effect on ectomycorrhizal fungi; Walker *et al*. 2011; Fujimura and Egger 2012; Timling *et al*. 2012; Botnen *et al*. 2014; Zhang and Yao 2015), indicating differential effects according to the fungal taxa (Abrego *et al*. 2020). Thus, further characterizations of plant-associated microbial communities are needed, particularly for some poorly studied Arctic regions, such as Norrbotten (Sweden), Iceland and Greenland (Denmark), Siberia and Novaya Zemlya (Russia), in order to better clarify the key drivers (i.e. host- and environmental-related factors) affecting the microbial community structure in the Arctic vascular plants.

## PLANT-ASSOCIATED MICROBIAL COMMUNITIES IN ANTARCTICA

Culture-independent studies showed that *D. antarctica* and *C. quitensis* host a wide taxonomic complexity of microbial communities (3–5 Shannon diversity index) at a similar level as that reported for plant-associated microbial communities in alpine and Arctic regions (Teixeira *et al*. 2010; Jorquera *et al*. 2016; Cid *et al*. 2017; da Silva *et al*. 2017; Kumar *et al*. 2017; Molina–Montenegro *et al*. 2019; Silva *et al*. 2020; Zhang *et al*. 2020). In particular, Proteobacteria, Actinobacteria, Firmicutes and Bacteroidetes were found as dominant bacterial phyla in leaves, roots and rhizosphere of Antarctica plants (Fig. 1 and Table 1; Teixeira *et al*. 2010; Cid *et al*. 2017; Molina–Montenegro *et al*. 2019; Silva *et al*. 2020; Zhang *et al*. 2020). Conversely, Acidobacteria, Choloroflexi and Verrucomicrobia were occasionally found (Silva *et al*. 2020; Zhang *et al*. 2020). In particular, *Pseudomonadaceae* was the most abundant family in both the endosphere and phyllosphere, whereas *Chitinophagaceae* dominated rhizosphere samples of *D. antarctica* and *C. quitensis* (Zhang *et al*. 2020), indicating plant tissue specificity of bacterial communities also in Antarctic regions (Fig. 2). Likewise, Pseudomonadales (*Pseudomonas* spp. and *Psychrobacter* spp.) and Rhizobiales (*Agrobacterium* spp. and *Aurantimonas* spp.) orders dominated *D. antarctica* phyllosphere (Cid *et al*. 2017), while *Bifidobacterium* (phylum Actinobacteria), *Arcobacter* (phylum Proteobacteria) and *Faecalibacterium* (phylum Firmicutes) genera dominated *D. antarctica* and *C. quitensis* rhizosphere (Teixeira *et al*. 2010). Culture-dependent approaches were used to investigate taxonomic structure of *D. antarctica* rhizosphere, and 72 psychrotolerant bacterial isolates were obtained (e.g. *Actinoplanes*, *Arthrobacter*, *Kribbella*, *Mycobacterium*, *Nocardia*, *Pilimelia*, *Pseudarthrobacter*, *Rhodococcus*, *Streptacidiphilus*, *Streptomyces* and *Tsukamurella* genera; Silva *et al*. 2020).

Ascomycota and Basidiomycota phyla dominated culturable fungal communities associated with *D. antarctica* and *C. quitensis* (Rosa *et al*. 2009, 2010; Santiago *et al*. 2012; Gonçalves *et al*. 2015; Martorell *et al*. 2017; Santiago, Rosa and Rosa 2017; Ferreira *et al*. 2019; Wentzel *et al*. 2019). For example, *Vishniacozyma victoriae* (formerly *Cryptococcus victoriae*) was the most abundant yeast associated with Antarctica plants (Vaz *et al*. 2011; Santiago, Rosa and Rosa 2017; Ferreira *et al*. 2019). Some fugal taxa were found in only one of the Antarctica plant species, suggesting host specificity of plant-associated microorganisms, such as *Rhodotorula mucilaginosa*, *Sporidiobolus ruineniae* and *Leucosporodium* aff. *golubevii* in *C. quitensis*, and *Cystobasidium laryngis* in *D. antarctica* (Santiago, Rosa and Rosa 2017). The occurrence of mycorrhizal fungi (e.g. *Glomus* spp. and *Acaulospora* spp.) and DSE (e.g. *Leptodontidium* spp., *Rhizoscyphus* spp., *Tapesia* spp. and *Mollisia* spp.) have also been found in Antarctica plants (Upson *et al*. 2009a;^8^; Barbosa *et al*. 2017; Hill *et al*. 2019) as possible ubiquitous plant-associated fungal taxa. However, some taxa were found exclusively in Antarctica plants and they were not reported in other environments (putative endemic microorganisms), such as *Antarctomyces* spp., *Dioszegia antarctica*, *Metschnikowia australis*, *Mrakia psychrophila* and *Naganishia antarctica* (formerly *Cryptococcus antarcticus*; Vaz *et al*. 2011; Arenz, Blanchette and Farrell 2014; Ferreira *et al*. 2019; Wentzel *et al*. 2019). Likewise, the bacterial genus *Clostridium* was found to include some endemic isolates of the Antarctic environments (Peixoto *et al*. 2016).

In Antarctica regions, plant species showed variable effect on the structure of microbial communities (Teixeira *et al*. 2010, 2013; Santiago, Rosa and Rosa 2017; Wentzel *et al*. 2019). Thus, additional factors can contribute to microbial community shaping, such as soil characteristics in the case of bacterial communities (Teixeira *et al*. 2010, 2013; Wentzel *et al*. 2019) or seasonal surface air temperature in the case of fungal communities (Upson, Newsham and Read 2008). However, plant-associated fungi in Antarctica environments have been mostly investigated using culture-dependent approaches and future culture-independent studies are required, in order to better assess taxonomic structure of fungal communities.

## ADAPTATION STRATEGIES OF PLANT-ASSOCIATED MICROBIAL COMMUNITIES TO HARSH CONDITIONS

Although meta-analyses are required to better highlight the existence of specific microbial taxa adapted to cold environments, some dominant bacterial taxa seems to be commonly present in alpine, Arctic, Antarctic regions, such as Proteobacteria, Actinobacteria and Bacteroidetes phyla, as well as Burkholderiales, Rhizobiales, Pseudomonadales, Bacillales, Actinomycetales (particularly *Microbacteriaceae*, *Micromonosporaceae* and *Micrococcaceae* families), Xanthomonodales, Saprospirales (particularly *Chitonophagaceae* family), Sphingobacteriales, Sphingomonodales and Myxococcales (Sheng *et al*. 2011; King *et al*. 2012; Nissinen, Männistö and van Elsas 2012; Angel *et al*. 2016; Jorquera *et al*. 2016; Cid *et al*. 2017; Kumar *et al*. 2017; Chica *et al*. 2019; Oberhofer *et al*. 2019; Praeg, Pauli and Illmer 2019; Given *et al*. 2020; Huang *et al*. 2020; Zhang *et al*. 2020). At low taxonomic level, *Bradyrhizobium*, *Burkholderia*, *Clavibacter*, *Clostridium, Flavobacterium*, *Micrococcus*, *Mycobaterium*, *Nocardia*, *Novosphingobium, Pedobacter*, *Pseudomonas*, *Rhizobium*, *Rhodoplanes*, *Sphingomonas* and *Streptomyces* genera dominates the plant-associated communities of alpine, Arctic and Antarctic regions (Sheng *et al*. 2011; Nissinen, Männistö and van Elsas 2012; Jorquera *et al*. 2016; Cid *et al*. 2017 Chica *et al*. 2019; Kumar *et al*. 2017; Oberhofer *et al*. 2019; Wassermann *et al*. 2019; Given *et al*. 2020; Huang *et al*. 2020; Ma *et al*. 2020; Zhang *et al*. 2020). Furthermore, members of these bacterial taxa were also shown to be cold-adapted and tightly associated with plants, suggesting their potential importance for plant fitness and survival in cold environments (King *et al*. 2012; Nissinen, Männistö and van Elsas 2012; Praeg, Pauli and Illmer 2019). Likewise, Ascomycota and Basidiomycota were the dominant phyla among plant-associated fungi in cold environments and some plant-associated fungal taxa can be frequently found in alpine, Arctic and Antarctic regions, such as *Cryptococcus*, *Fusarium*, *Mrakia* and *Rhodotorula* genera (Ferreira *et al*. 2019; Li *et al*. 2018; Praeg, Pauli and Illmer 2019; Santiago, Rosa and Rosa 2017; Wassermann *et al*. 2019; Zhang and Yao 2015). Although some plant-associated microbial taxa have a global distribution, their relative abundance and taxonomic complexity seemed to be higher in cold environments compared to benign regions, such as mycorrhizal fungi in alpine regions (Acaulosporaceae; e.g. *Acaulospora alpina* and Ambisporaceae; e.g. *Ambispora fennica* families; Oehl *et al*. 2006; Liu *et al*. 2011; Li *et al*. 2014; Senés-Guerrero and Schüssler 2016; Yang *et al*. 2016; Casazza *et al*. 2017; 2018; Haug, Setaro and Suárez 2019) and Arctic regions (e.g. *Thelephora*, *Tomentella, Inocybe*, *Cortinarius* and *Cenococcum* genera; Bjorbaekmo *et al*. 2010; Timling *et al*. 2012) or *Psychrobacter* and *Exiguobacterium* genera in Arctic and Antarctic regions (Rodrigues and Tiedje 2007; Rodrigues *et al*. 2009; Cid *et al*. 2017). However, no large comparative studies have been conducted on plant-associated microbial communities across different cold environments, indicating that further quantitative studies are needed to confirm the existence of cold-adapted microbial taxa consistently associated to plants in cold environments. For example, some plant species (e.g. *Bistorta*, *Diapensia*, *Dryas*, *Juncus*, *Oxyria* and *Saxifraga*) are widely distributed in both alpine and Arctic regions and they could be suitable for a comparative analysis of microbial communities associated to the same host in different cold regions (Fisher *et al*. 1995; Bjorbækmo *et al*. 2010; Davey *et al*. 2015; Kumar *et al*. 2017; Botnen *et al*. 2019). In particular, Kumar and colleagues (2017) investigated the bacterial community structure of *O. digyna* and *S. oppositifolia* in three different climatic regions (alpine, low Arctic and high Arctic), and found that both plants shared bacterial taxa (core microbiota) belonging to Burkholderiales, Actinomycetales and Rhizobiales.

Functional studies were carried out on plant-associated microorganisms of cold regions, suggesting possible adaptation strategies to the harsh conditions. For example, functional studies of plant-associated bacteria in Arctic regions demonstrated that rhizosphere communities of *O. digyna* and *S. oppositifolia* tolerate oxidative stress and produce antibiotic molecules (e.g. fusidic acid, surfactant Niaproof 4 and troleandomycin; Kumar *et al*. 2016). Cold treatments can also upregulate genes involved in sugar transport, protein transport, lipid biosynthesis and NADH oxidoreductase activity, as demonstrated by the transcriptional profiling of an Arctic *Mesorhizobium* strain N33, isolated from nodules of *Oxytropis arctobia* in Canada (Ghobakhlou *et al*. 2015). Moreover, genomic studies indicated the presence of possible adaptation strategies of plant-associated microorganisms to cold environments, as revealed by the presence of genes encoding ice-nucleation proteins in *Pseudomonas* isolates of Antarctica plants (Cid *et al*. 2018) and genes related to cold stress response, membrane transport and osmotic regulation in cold tolerant *Bacillus* spp. (Zubair *et al*. 2019). In addition, genes involved in the utilization of various carbon sources and production of antibiotics, phytohormones, pigments and antioxidants were found in microbial communities associated with *Espeletia* plants in alpine regions (Ruiz-Pérez, Restrepo and Zambrano 2016), suggesting an efficient nutrient acquisition and a strict microbe-host interaction.

Heterotrophy, fermentation, xenobiotic degradation, nitrogen metabolism, tryptophan metabolism and inositol metabolism were the major functional groups of plant-associated microbial communities in Antarctic regions (Peixoto *et al*. 2016; Zhang *et al*. 2020), indicating a possible adaptation strategy to harsh environmental conditions. Some other functions were found in plant-associated communities of alpine regions according to the host tissue, such as streptomycin production, biomass degradation, xylan degradation and carbon fixation in rhizosphere communities; and nitrite reduction, ammonia oxidation and chitin degradation in endosphere communities (Huang *et al*. 2020). Comparative and functional metagenomic analysis of rhizosphere microorganisms associated with *C. quitensis*, either growing alone or together with *D. antarctica*, revealed differences in the abundance of genes related to environmental tolerance, cellular metabolism and osmotic stress, suggesting that such microorganisms could display specific functional activities that could have an effect on plant colonization and environmental tolerance (Molina-Montenegro *et al*. 2019).

The above-mentioned examples indicate that plant-associated microbial communities in cold environments may have developed various adaptation strategies for cold stress tolerance, including genetic features that enables them to perform metabolic and physiological functions under cold conditions Although some studies were able to predict biological functions of microbial communities using taxonomic or genomic information, further functional analyses are required to clarify cellular mechanisms of microbial tolerance to harsh environmental conditions. Moreover, the integration of multiomic approaches (e.g. genomics, metagenomics, transcriptomics, proteomics and metabolomics) will also help to better understand microbial adaptation strategies in cold environments by identifying key genes, proteins and metabolites whose regulation is affected by cold temperatures.

## POTENTIAL FUNCTIONS OF PLANT-ASSOCIATED MICROORGANISMS FROM COLD REGIONS

To mitigate the effect of climate changes in agriculture, new ecofriendly and sustainable strategies are needed, particularly to limit negative effects of cold stress on crops. In particular, global warming is expected to promote earlier spring-related phenological events in plants, and, as a consequence, it will increase the risk and severity of spring frosts (Menzel *et al*. 2006; Gu *et al*. 2008). The use of plant-associated microorganisms and their compounds could be one of the most promising solution against cold stresses, but most microbial products for crop protection commonly used in agriculture are based on mesophilic microorganisms, which are unable to exert positive effects on plant growth in cold conditions (Wu *et al*. 2019; Torracchi *et al*. 2020). Conversely, plant-associated microorganisms isolated from cold environments, including bacteria (e.g. *Bacillus* spp., *Brevibacterium* spp., *Clavibacter* spp. and *Pseudomonas* spp.) and fungi (e.g. *Geomyces* spp., *Lecanicillium* spp. and *Neotyphodium* spp.; mycorrhizal fungi: *Glomus* spp.; and DSE: *Mollisia* spp., *Phialocephala* spp. and *Tapesia* spp.), could be used to promote plant growth under cold stress (Upson, Read and Newsham 2009b; Haselwandter and Read 1982; Ruotsalainen and Kytoviita 2004; Wäli *et al*. 2008; Ding *et al*. 2011; Mishra *et al*. 2011; Berríos *et al*. 2013; Suyal, Shukla and Goel 2014; Molina-Montenegro *et al*. 2015; Yarzábal *et al*. 2018; Hill *et al*. 2019; Tiryaki, Aydın and Atıcı 2019; Wu *et al*. 2019; Zubair *et al*. 2019; Tapia-Vázquez *et al*. 2020). Some other microorganisms isolated from cold environments (e.g. *Achromobacter*, *Enterobacter*, *Exiguobacterium*, *Pseudomonas*, *Rahnella* and *Stenotrophomonas*) promoted plant growth under normal conditions (16–25°C), although their effect on cold-stressed plants was not yet investigated (Selvakumar *et al*. 2009, 2011; Vyas *et al*. 2010; Ghyselinck *et al*. 2013; Ogata-Gutiérrez *et al*. 2017; Castellano-Hinojosa *et al*. 2018; Chumpitaz-Segovia *et al*. 2020). For example, 37 psychrophilic isolates of *Bacillus* spp. (including *B. pumilus*, *B. safensis* and *B. atrophaeus* able to grow at below 10°C) obtained from the plant rhizosphere in the Qinghai-Tibetan plateau (2788–4780 m a.s.l.) promoted the growth of winter wheat seedlings at 10°C (Wu *et al*. 2019). Likewise, *Pseudomonas*, *Bacillus* and *Enterobacter* isolates obtained from native potato varieties in the high Andes promoted plant growth, but also reduced *Rhizoctonia solani* severity (Ghyselinck *et al*. 2013). Root-associated microorganisms positively influenced *Plantago major* growth in a plant ecotype-dependent manner: plant ecotypes growing with their local root-associated microorganisms performed better than when growing with foreign microorganisms (Formenti *et al*. 2020). Thus, the development of microbial biostimulants based on indigenous cold-adapted microorganisms could be a promising approach to protect crop plants from cold stress, but further functional studies are required to better characterize the modes of action and possible limitations due to host specificity of psychrotolerant plant-growth promoting bacteria.

Plant growth promoting traits (e.g. production of indole-3-acetic acid, gibberellic acid, 1-aminocyclopropane-1-carboxylate-deaminase, hydrogen cyanide, hydrogen sulfide, ammonia, siderophores, hydrolytic enzymes, phosphate solubilizing products and nitrogen fixing activity) were demonstrated in numerous microorganisms isolated from alpine (Calvo *et al*. 2010; Sheng *et al*. 2011; Wang *et al*. 2016; Yadav *et al*. 2016; Ogata-Gutiérrez *et al*. 2017; Castellano-Hinojosa *et al*. 2018; Chumpitaz-Segovia *et al*. 2020; Ma *et al*. 2020; Tapia-Vázquez *et al*. 2020; Ulloa-Muñoz *et al*. 2020; Yang *et al*. 2020), Arctic (Nosko, Bliss and Cook 1994; Sun *et al*. 1995) and Antarctic environments (Barrientos-Díaz, Gidekel and Gutiérrez-Moraga 2008; Selvakumar *et al*. 2011; Berríos *et al*. 2013; Peixoto *et al*. 2016; Yarzábal *et al*. 2018; Tiryaki, Aydın and Atıcı 2019; Tistechok *et al*. 2019; Araya *et al*. 2020). Some microbial traits were also implicated in the improvement of cold stress tolerance of plants, such as antioxidant (e.g. superoxide dismutase and peroxidases), osmolyte (e.g. trehalose and raffinose) and nutrient (e.g. nitrogen and phosphorus) production (Acuña-Rodríguez *et al*. 2020). However, the majority of studies have investigated the functional role of plant-associated microorganisms from cold environments on plant growth and tolerance to cold stress under controlled conditions, and further validations of their effects under field conditions are needed for the further development of cold stress protecting agents based on psychrotolerant plant-growth promoting bacteria.

Besides the protection of plants against cold stress, many plant-associated microorganisms isolated from cold environments can produce a wide-variety of bioactive compounds that could be biotechnologically relevant for the industry and medicine (Barrientos-Díaz, Gidekel and Gutiérrez-Moraga 2008; Rosa *et al*. 2010; Vaz *et al*. 2011; Santiago *et al*. 2012; Vasileva-Tonkova *et al*. 2014; Cui *et al*. 2015; Gonçalves *et al*. 2015; Cid *et al*. 2016; Lamilla *et al*. 2017; Martorell *et al*. 2017; Pandey *et al*. 2018; Wentzel *et al*. 2019; Jain *et al*. 2020; Silva *et al*. 2020). For example, cold-active enzymes (e.g. cellulase, gelatinase, lipase, ligninolytic, phosphatase and protease), ice-binding proteins (e.g. antifreeze and ice-nucleation proteins), natural pigments (e.g. carotenoids and melanin) and antioxidants compounds (e.g. flavonoids and phenolic compounds) were identified in plant-associated microorganisms from cold environments and are available for further industrial applications (Barrientos-Díaz, Gidekel and Gutiérrez-Moraga 2008; Rosa *et al*. 2010; Vaz *et al*. 2011; Vasileva-Tonkova *et al*. 2014; Cui *et al*. 2015; Cid *et al*. 2016; Lamilla *et al*. 2017; Pandey *et al*. 2018; Wentzel *et al*. 2019; Jain *et al*. 2020). Moreover, plant-associated fungi and bacteria from the Antarctica showed leishmanicidal, trypanocidal and antitumoral activities (Santiago *et al*. 2012; Gonçalves *et al*. 2015) for a further pharmaceutical development, such as cinerubin B and actinomycin V produced by two Antarctic *Streptomyces* strains (CMAA 1527 and CMAA 1653) against human cancer cells (Silva *et al*. 2020).

## CONCLUSIONS

Plants growing in cold environments harbor complex, host-specific and cold-adapted microbial communities that may play key functional roles in plant growth and survival. However, most studies investigated the taxonomic structure and potential functions of plant-associated bacterial and fungal communities in cold environments, while deeper taxonomic and functional studies are required on plant-associated Archaea and protists. Although microbial communities of plant rhizosphere and vegetative organs (e.g. roots, stems and leaves) were studied, taxonomic structure and functional properties of those associated with reproductive organs (e.g. flowers, fruits and seeds) needs further investigations. Future comparative studies and meta-analyses on plant microbiota in cold and temperate environments are also required, in order to identify possible microbial taxa specifically adapted to cold environments.

## ACKNOWLEDGMENTS

The authors apologize to the scientists that are not cited because of space limitation.
